# Public health round-up

**DOI:** 10.2471/BLT.23.010423

**Published:** 2023-04-01

**Cholera in the Syrian Arab Republic**

People shelter in a stadium in Aleppo in the Syrian Arab Republic. Around 90 000 people have taken refuge in camps and reception centres in the north-west of the country in the aftermath of the devastating earthquakes that struck southern Türkiye and northern Syrian Arab Republic on 6 February. The quakes disrupted access to safe water, sanitation, and hygiene, exposing the population to an increased risk of waterborne diseases, including cholera. A cholera vaccination campaign was launched by the World Health Organization (WHO) and partners in coordination with local health authorities on 8 March.

**Figure:**
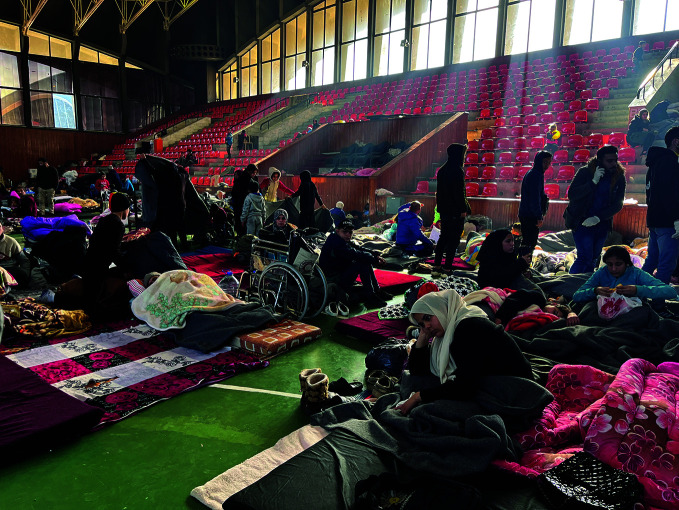
WHO/Inas Hamam

## Syrian Arab Republic emergency response

WHO and the United Nations Children’s Fund, in coordination with health authorities, the Syria Immunization Group, and Gavi, the Vaccine Alliance, launched a cholera vaccination campaign in earthquake-hit areas in the north-west of the Syrian Arab Republic.

Announced on 8 March, the 10-day campaign was set to deliver 1.7 million doses of cholera vaccine to protect Syrians above one year of age, especially those living in the areas most severely impacted by the earthquakes and at highest risk of cholera exposure.

WHO warned of increased risk of waterborne diseases for over 2.1 million Syrians, especially those living in overcrowded displaced persons camps. As of 28 February, 92 649 suspected cases of cholera had been reported in the country, half in the north-west.

In related news, WHO Director-General Tedros Adhanom Ghebreyesus visited the north-west of the country on 1 March, meeting with the partners who are working with WHO to deliver essential health care, including specialized orthopaedic and paediatric care.

As of 1 March, WHO had sent over 140 metric tonnes of supplies to the Syrian Arab Republic from across the border in Türkiye, and in the first hours after the earthquakes was able to distribute 183 metric tonnes of supplies prepositioned in warehouses in Azaz and Idlib in the north-west of the country to more than 200 health facilities.

http://bit.ly/3ZMmGWM

http://bit.ly/3T9JZI3

## Marburg in Equatorial Guinea

Public health authorities in Equatorial Guinea initiated epidemiological investigations to determine the source of the country’s first outbreak of Marburg virus disease. National teams were deployed to the affected districts to conduct active case finding and contact tracing, while isolating and providing medical care to people infected.

As of 21 February, nine cases had been reported, including one confirmed, four probable and four suspected cases. All the patients concerned had died, one in a health facility and the other eight in the community. There were no cases among health-care workers. Thirty-four contacts were being followed up.

Investigations were ongoing to find additional cases, and WHO was providing support by strengthening contact tracing, case management, infection prevention and control, laboratory, risk communication and community engagement. WHO assessed the risk posed by the outbreak to be high at the national level, moderate at the regional level and low at the global level.

http://bit.ly/3YizTWg

## Avian Influenza A in Cambodia

An investigation was launched into two cases of avian influenza A (H5N1) virus infection in Cambodia. The first case was reported on 23 February by the Cambodia International Health Regulations National Focal Point. The following day, a family member in contact with the first person to be infected was also reported to be infected. These were the first human H5N1 infections to be reported in Cambodia since 2014.

Human H5N1 infection can cause severe disease and has a high mortality rate. The available evidence suggests that current avian influenza A (H5) viruses have not acquired the ability of sustained transmission among humans, thus the likelihood of sustained human-to-human spread in this case is assessed to be low.

As of 26 February, analysis of the virus from the two infections was ongoing. An outbreak investigation had also been launched, while close contacts of the two people infected were being monitored.

http://bit.ly/3YeGQY8

## WHO tackling sexual misconduct

WHO launched a new policy on preventing and addressing sexual misconduct. Launched on 3 March, the policy replaces a 2017 policy, places the victims and survivors at its core, and sets strict standards of zero tolerance.

The policy covers WHO staff and collaborators (consultants, contractors, partners) in locations where WHO operates, and sets six minimum standards to protect anyone subject to sexual misconduct by WHO staff or collaborators.

WHO Director-General, Tedros Adhanom Ghebreyesus said the new policy builds on the work already done in implementing the recommendations of the independent commission that he established in 2020 to investigate allegations of sexual exploitation and abuse during the response to the 10^th^ Ebola virus disease outbreak in the Democratic Republic of the Congo. He said that the policy is key to making zero tolerance “a reality and not merely a slogan.”

In related news, WHO’s Regional Director for the Western Pacific was dismissed after allegations of misconduct were upheld in a review process which included consultation with the Regional Committee for the Western Pacific and the Executive Board.

http://bit.ly/3L6acFp

http://bit.ly/420T8qk

## Toxic skin-lightening cosmetics

The governments of Gabon, Jamaica and Sri Lanka launched a joint 14 million United States dollars project to tackle the issue of mercury use in skin-lightening products.

Announced on 14 February, the three-year project will work to reduce the risk of exposure to mercury-added skin-lightening products, raise awareness of the health risks associated with their use, and address cultural norms relating to skin complexion by engaging organizations, health-care professionals and influencers working in the field.

The 2013 Minamata Convention on Mercury sets a limit of 1milligram per kilogram for mercury in skin-lightening products, but a recent study of over 300 products from 22 countries found that one in ten products exceeded this limit, with many containing up to 100 times the authorized amount.

http://bit.ly/3J3oHIy

## Preparing for the next pandemic

WHO Member States began negotiations on a global accord to protect nations and communities from future pandemic emergencies. Discussions based on a preliminary draft pandemic accord took place during the week-long fourth meeting of the Intergovernmental Negotiating Body (comprised of representatives from WHO’s 194 Member States) which ended on 3 March.

Negotiations on the draft will continue over the next year according to a timetable set by the World Health Assembly, with a view to producing a final draft for consideration by the seventy-seventh World Health Assembly in 2024.

http://bit.ly/3yqZHVN

**Cover photo**

A doctor examines a baby at the *Centre médico-social pour la protection maternelle et infantile de Montravel*, in Nouméa, New Caledonia.

**Figure:**
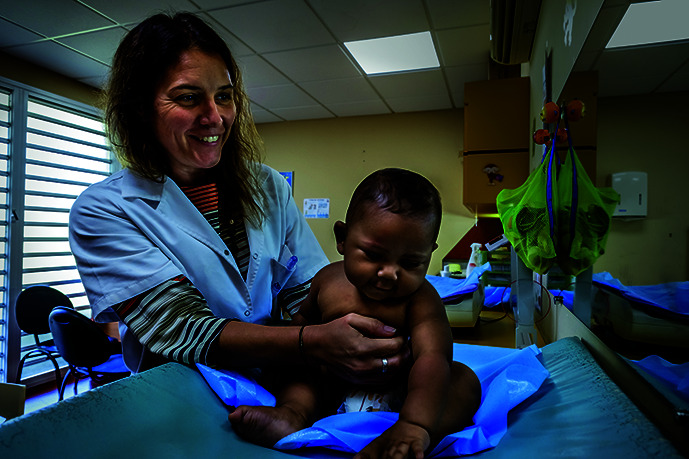
WHO/Yoshi Shimizu

## Reducing sodium intake

Only nine countries worldwide (Brazil, Chile, Czechia, Lithuania, Malaysia, Mexico, Saudi Arabia, Spain and Uruguay) have introduced comprehensive packages of recommended policies to reduce sodium intake.

This is according to WHO’s inaugural *Global report on sodium intake* *reduction*, which shows that the world is off-track to achieve its global target of reducing sodium intake by 30% by 2025.

Released on 9 March, the report is designed to support the monitoring of progress and identify areas for action, introducing a Sodium Country Score from 1 (the lowest level) to 4 (the highest level) which can be used to assess the impact of policy on population dietary sodium intake and cardiovascular disease.

While an essential nutrient, sodium increases the risk of heart disease, stroke and premature death when eaten in excess. The main source of sodium is table salt, but it is also contained in other condiments such as sodium glutamate. By implementing highly cost-effective sodium reduction policies, it is estimated that some 7 million lives could be saved globally by 2030.

http://bit.ly/3F8Q819

## Holistic monitoring of child health

WHO launched a new set of measures to monitor the development of children up to three years of age at population level. Launched on 27 February, the Global Scales for Early Development (GSED) allow for a comprehensive assessment of the development of young children, capturing cognitive, socio-emotional, language and motor skills.

The GSED provides a new common unit to measure development known as a developmental score (D-score), allowing for the generation of a holistic picture of children’s development which can be tracked over time.

The aim of the measures is to help countries, programmes and researchers gather and use data on early childhood development, to better inform investment in the services and support needed.

http://bit.ly/3EOTs1m

**Looking ahead**

3–5 April 2023. Fifth Global Forum on Human Resources for Health. Geneva, Switzerland. http://bit.ly/3kjKQsm

24–26 April 2023. The Preparedness and Resilience for Emerging Threats Global Meeting. Geneva, Switzerland. http://bit.ly/3ZLGPMH

4–5 May 2023. Global Human and Veterinary Medicines Regulatory Authorities Summit and Forum to Preserve Antimicrobials. Geneva, Switzerland. http://bit.ly/3L5Pllr

